# Stability of Capsaicinoids and Antioxidants in Dry Hot Peppers under Different Packaging and Storage Temperatures

**DOI:** 10.3390/foods4020051

**Published:** 2015-03-31

**Authors:** Qumer Iqbal, Muhammad Amjad, Muhammad Rafique Asi, Agustin Ariño, Khurram Ziaf, Aamir Nawaz, Tanveer Ahmad

**Affiliations:** 1 Institute of Horticultural Sciences, University of Agriculture, Faisalabad 38000, Pakistan; E-Mails: amjaduaf@gmail.com (M.A.); khurramziaf@uaf.edu.pk (K.Z.); horticulture.tanveer@gmail.com (T.A.); 2 Nuclear Institute for Agriculture and Biology (NIAB), Faisalabad 38000, Pakistan; E-Mail: asimuhammad@yahoo.co.uk; 3 Veterinary Faculty, University of Zaragoza, Zaragoza 50013, Spain; E-Mail: aarino@unizar.es; 4 Faculty of Agricultural Sciences and Technology, Bahauddin Zakariya University, Multan 60000, Pakistan; E-Mail: aamirkhan_74@hotmail.com

**Keywords:** hot peppers, capsaicinoids, total carotenoids, ascorbic acid, total phenolics, storage

## Abstract

The maintenance of the quality and storage life of perishable fruits and vegetables is a major challenge for the food industry. In this study, the effects of different temperatures, packaging materials and storage time on the stability of capsaicinoids and antioxidants, such as total carotenoids, ascorbic acid and total phenolic compounds, were studied in three commercially cultivated hot pepper hybrids, namely Sky Red, Maha and Wonder King. For this purpose, dry whole pods were packed in jute bags and low-density polyethylene bags (LDPE), stored for five months under controlled conditions at 20, 25 or 30 ^○^C and analyzed on Day 0 and at 50-day intervals until Day 150. The three hot pepper hybrids differed significantly with respect to their capsaicinoids and antioxidant concentrations, but the results indicated that with the increase in storage temperature and time, a gradual and steady decrease in these levels was equally observed for all hybrids. Overall, mean concentrations after five months were significantly reduced by 22.6% for ascorbic acid, 19.0% for phenolic compounds, 17% for carotenoids and 12.7% for capsaicinoids. The trends of capsaicinoids and antioxidants evolution were decreasing gradually during storage until Day 150, this effect being more pronounced at higher temperature. Furthermore, the disappearance rates of capsaicinoids and antioxidants were higher in peppers packed in jute bags than in those wrapped with LDPE. In conclusion, despite the sensitivity of capsaicinoids and antioxidants to oxygen, light and moisture, the packaging in natural jute or synthetic LDPE plastic bags, as well as the storage at ambient temperature preserved between 77.4% and 87.3% of the initial amounts of these health- and nutrition-promoting compounds during five months’ storage.

## 1. Introduction

Pepper fruits (*Capsicum annuum*) are important vegetables consumed worldwide as food and as spice condiments, such as curry, chili powder and paprika. The dehydrated fruits in the red ripe stage are globally commercialized and valued for their nutritional, health and sensory attributes. Peppers are regarded to be a good source of nutrients and phytochemicals, such as ascorbic acid, carotenoids and phenolic compounds, with well-known antioxidant properties and potential health benefits [1,2]. Hot cultivars are rich in capsaicinoids, responsible for the specific taste of pepper fruits, which may be also used in pain relievers, due to their pharmacological properties [3]. Additionally, the presence of capsaicinoids in hot peppers has a favorable effect on the stability of carotenoids during thermal drying [4]. The amounts of bioactive compounds depend on the pepper variety, ripening stage and growing conditions. A recent study conducted in the present authors’ laboratory showed a considerable increase in ascorbic acid and carotenoid contents during the course of ripening in hot peppers [5].

Although there seems to be a general agreement about the bioactive compounds present in hot peppers, there is little information available about the stability of capsaicinoids and antioxidants during postharvest storage and the consequences for their nutritional value and contribution to health. A number of works have reported on the effect of storage and packing materials on the long-term stability of polyphenols in foods and drinks and noted that they are very sensitive to light, oxygen and heat [6,7]. Talcott *et al*. [8] revealed that the antioxidant property of carrot polyphenols greatly reduced during storage at high temperatures, while Mustapha and Selselet-Attou [9] observed significant reduction in total phenolic contents in dates even at 10 °C after five months’ storage. Several earlier studies focused on the stability of capsaicinoids and antioxidants in paprika stored at various temperatures and reported substantial fluctuations during storage. Thus, the concentration of capsaicinoids in paprika was significantly decreased as storage prolonged under ambient storage conditions, and the maximum decrease was recorded for dihydrocapsaicin [10]. Similarly, ascorbic acid concentration in ground paprika decreased significantly by 35% of the original level after four months of storage [11], while carotenoids and flavonoids showed more stability [12]. The evolution of carotenoid content in paprika powder indicated that 83% of the original concentration was retained in product stored in plastic sacs at 4 °C during 12 months [13].

However, analyses of the stability of capsaicinoids and antioxidants in paprika powder cannot be used to draw parallels with whole fruits, since processing conditions may alter enzyme-catalyzed processes, whereas postharvest ripening continues to take place in dry fruits during storage. In Pakistan, dry hot peppers are usually stored in jute bags at retail stores, but in mega stores and trading centers, they are available in polyethylene bags. Storage conditions and time after harvest have a great impact on the bioactive compounds of any produce. Therefore, the objective of the present work was to study the stability of capsaicinoids and antioxidants of dry whole hot peppers in protective packaging of polyethylene or jute during storage for five months at selected conditions of temperature, applying those normally used by hot pepper dealers.

## 2. Experimental Section

### 2.1. Pepper Samples 

Three commercially-available hot pepper hybrids (*Capsicum annuum* L.), namely Sky Red, Maha and Wonder King, were grown in plastic tunnels under a drip irrigation system at the Vegetable Research Area of the Institute of Horticultural Sciences (University of Agriculture, Faisalabad, Pakistan) and harvested at the red ripe stage with pedicels. The fruits were dried in the sun for 6 to 8 days with an average daily temperature of 39 °C and relative humidity of 36% before the storage experiments. The moisture content of dried samples was determined with the air-forced oven drying method (indirect distillation at 105 ^○^C), according to Method 44-15A of the American Association for Cereal Chemistry [14]. Moisture content in all hot pepper samples was 12%–13% after the sun-drying period.

### 2.2. Packaging and Storage Conditions

Dried pods of hot pepper hybrids (250 g) were packed in either natural jute bags or synthetic low-density polyethylene bags (9 µm thick, 20 by 32 cm). All samples were stored for 5 months in humidifiers (National MFG. Co., Lincoln, NE, USA) under controlled conditions at 20, 25 or 30 ^○^C with 75% relative humidity, and analyzed on Day 0 and at 50-day intervals until Day 150. Experiments were conducted in triplicate.

### 2.3. Capsaicin and Dihydrocapsaicin

Hot pepper pods were oven-dried at 60 °C for 2–5 days, cooled and then ground to dried pepper powder. Samples were analyzed by using a chromatographic method previously described [5]. A mixture of sample:acetonitrile in a ratio of 1:10 was placed in 120-mL glass bottles with Teflon-lined lids, capped and placed in a water bath at 80 °C for 4 h and swirled manually every hour. The bottles were removed from the water bath and cooled at room temperature. The supernatant content of samples (2–3 mL) was filtered through a 0.45 μm filter (Millex^®^-HV filter) using a 5-mL disposable syringe (Millipore, Bedford, MA, USA) into an HPLC sample vial. For the liquid chromatographic analysis of capsaicinoids, an HPLC system LC-10 (Shimadzu, Japan) equipped with an SPD-10A UV-Vis detector (set at 280-nm wavelength) was used. The analysis was carried out with the isocratic mobile phase (acetonitrile:water, 60:40) at a flow rate of 1 mL/min using a column Discovery C18 (250 × 4.6 mm, 5 μm) supplied by Supelco (Bellefonte, PA, USA). The limit of detection (LOD) was 0.1 µg/g for both capsaicinoids.

### 2.4. Total Carotenoids

Based on the Association of Official Analytical Chemists (AOAC) official Method 970.64 [15], two grams of hot pepper sample were ground using a mortar and pestle and transferred to a 100-mL flask covered with a stopper. The sample was blended for one minute with a mixture of 30 mL hexane:acetone:ethanol:toluene (10:7:6:7). For hot saponification, 2 mL of 40% methanolic KOH were pipetted into the flask, swirled for one minute and placed in a 56 °C water bath for 20 min. The sample was cooled for one hour in the dark, and then, 30 mL of hexane were pipetted into the flask, dried over anhydrous sodium sulfate made up to volume and shaken vigorously for one minute. Upper phase was 50 mL. Absorbance was measured at 436 nm using the IRMECO UV-Vis spectrophotometer Model U2020 with β-carotene (Sigma-Aldrich, St. Louis, MO, USA) as the standard.

### 2.5. Ascorbic Acid 

Ascorbic acid was quantitatively determined according to the 2,6-dichlorophenolindophenol AOAC official Method 967.21 [16]. A sample of hot peppers (10 g) was blended with 2.5 mL of 20% metaphosphoric acid, and distilled water was then added up to the 100-mL mark. Ten milliliters of the suspension were titrated with freshly-prepared standard of 2,6-dichlorophenolindophenol dye until a light, but distinct rose pink color persisted for 15 s.

### 2.6. Total Phenolic Compounds 

Total phenolic contents of hot peppers were analyzed using the modified Folin-Ciocalteu reagent method as described elsewhere [5]. About 0.5 g of the sample was macerated in 3 mL 80% aqueous acetone with a mortar and pestle. The extracts were placed into tightly-sealed micro-tubes and maintained in darkness at 4 °C overnight. Samples were centrifuged at 1000 rpm for 2 min. A mixture of 135 μL H_2_O, 750 μL 1/10 dilution Folin-Ciocalteu reagent (Sigma-Aldrich, St. Louis, MO, USA) and 600 μL 7.5% (w/v) Na_2_CO_3_ was added to 50 μL of extract in 1.5-mL micro-tubes. After vortexing for 10 s, the mixture was incubated at 45 °C in a water bath for 15 min. Samples were allowed to cool to room temperature before reading the absorbance at 765 nm using the IRMECO UV-Vis spectrophotometer Model U2020. A blank was prepared from 50 μL 80% aqueous acetone. The gallic acid standard curve was prepared from freshly-made 1 mg/mL gallic acid (Acros Organics, Belgium) in 80% aqueous acetone.

### 2.7. Statistical Analysis

Analysis of variance was computed with the data from each attribute using the STATISTICA Computer Program (Version 2003, StatSoft Inc., Tulsa, OK, USA). The least significant difference test at the 5% level of probability was used to check the differences among mean values according to Hill and Lewicky [16]. There were 72 treatments, and each treatment was replicated thrice.

## 3. Results and Discussion

### 3.1. Evolution of Capsaicinoids Contents 

**Figure 1:**
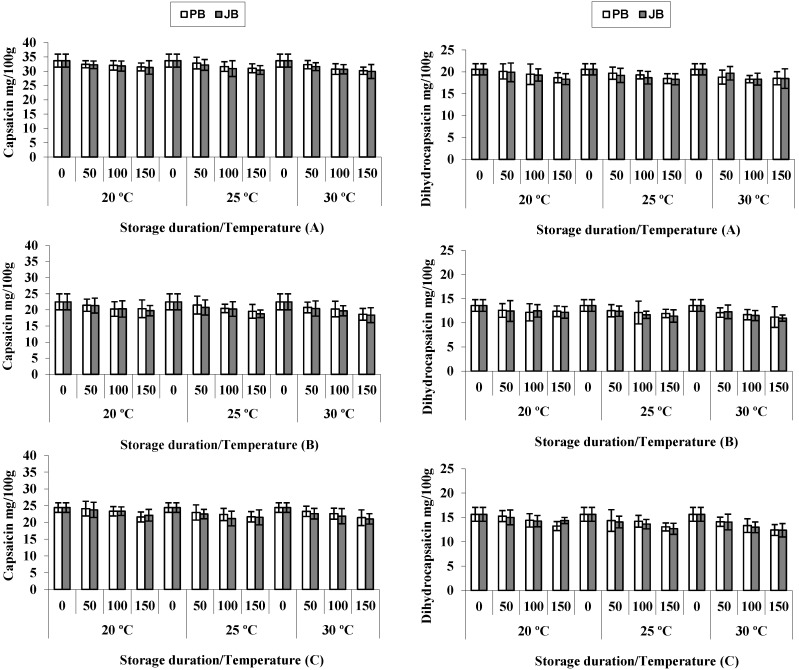
Capsaicin and dihydrocapsaicin concentrations in hot peppers *viz*. Sky Red (**A**), Maha (**B**) and Wonder King (**C**) at different temperatures and packaging materials (PB, polyethylene bag; JB, jute bag) during five months’ storage. Vertical bars show ± SD of *n* = 3 replicates.

Pungency is an important factor for determining the commercial quality in hot peppers, the level of which depends mainly on the concentration of capsaicinoids, such as capsaicin and dihydrocapsaicin [17]. The former is the primary molecule responsible for the heat and burning sensations associated with hot peppers. The initial levels of capsaicin and dihydrocapsaicin were significantly different among hybrids (Table 1), Sky Red (33.7 and 20.6 mg/100 g), Maha (22.5 and 13.6 mg/100 g) and Wonder King (24.4 and 15.6 mg/100 g), respectively (Figure 1). Overall, capsaicin concentrations in polyethylene-packed hot peppers gradually decreased during 150-day storage by 8.8% at 20 °C, 10.4% at 25 °C and 12.9% at 30 °C, while decreasing amounts observed in samples packed in jute bags were 9.2%, 12.2% and 14.0%, respectively (Table 2). Dihydrocapsaicin evolved in a similar trend showing a reduction of 11.1%, 12.8% and 15.4% (20 °C, 25 °C and 30 °C, respectively) in hot peppers packed in polyethylene bags after 150 days of storage, whereas for those stored in jute bags, the percent reductions reached 10%, 14.9% and 16% (Table 2). Then, both capsaicinoids decreased significantly during storage for 150 days (*p <* 0.05), and the disappearance rate increased with storage temperature and tended to be higher in jute bags than in polyethylene wrapping. Jute is a natural fiber that is permeable to air and moisture, and it is biodegradable and easily decomposable in the environment. In contrast, polyethylene is a low cost and low weight, durable, synthetic polymer that provides a barrier to oxygen and water vapor, but it is not biodegradable and causes pollution of the environment.

The trends of capsaicinoids evolution in the three hot peppers hybrids can be observed in Figure 1. The highest reductions of capsaicin were observed in the Maha hybrid, followed by Wonder King and Sky Red, whereas the rate of dihydrocapsaicin disappearance was highest in Wonder King, followed by Maha and Sky Red. These results indicated that the three hot peppers hybrids behave somewhat differently with respect to the fading out of capsaicinoids during storage. It has been reported that differences in biochemical factors (*i.e*., peroxidase activity) within hot pepper hybrids can influence the stability of capsaicinoids with the advancement of maturity and drying [18].

Overall, our studies indicated that with the increase in storage temperature and time, a gradual and steady decrease in capsaicinoids concentrations was observed in hot peppers. Available research information regarding the effect of temperature and packaging materials on capsaicinoids concentrations is scarce and mainly focused on powder paprika. In correlation with our results, Topuz and Ozdemir [10] observed 16% and 15% decrease in capsaicin and dihydrocapsaicin concentrations, respectively, in powder paprika after six months’ storage at ambient temperature.

**Table 1:** Mean square values from the analysis of variance for capsaicin, dihydrocapsaicin, total carotenoids, ascorbic acid and total phenolic contents (mg/100 g) of hot pepper hybrids under different packaging and storage conditions.

Source of variation	Degree freedom	Capsaicin	Dihydrocapsaicin	Total carotenoids	Ascorbic acid	Total phenolic compounds
Hybrid (H)	2	2,537.866 **	937.711 **	16,090.310 **	1,068.297 **	13,753.680 **
Storage Period (S)	3	87.174 **	51.529 **	2,306.240 **	845.790 **	1,064.536 **
Packaging (P)	1	4.544 **	1.123 *	78.313 **	39.681 **	22.633 **
Temperature (T)	2	9.435 **	6.982 **	276.108 **	223.127 **	143.317 **
H × S	6	0.461 *	0.749 **	21.130 **	15.002 **	62.977 **
H × P	2	0.007 ^NS^	0.020 ^NS^	5.870 ^NS^	0.292 ^NS^	0.176 ^NS^
H × T	4	0.472 *	0.127 ^NS^	10.700 *	8.648 **	4.276 **
S × P	3	0.696 **	0.226 ^NS^	13.888 *	4.884 **	3.217 **
S × T	6	1.215 **	0.874 **	76.090 **	44.643 **	18.147 **
P × T	2	0.475 ^NS^	0.257 ^NS^	15.352 *	0.592 ^NS^	1.139 ^NS^
H × S × P	6	0.066 ^NS^	0.031 ^NS^	1.517 ^NS^	0.619 ^NS^	0.214 ^NS^
H × S × T	12	0.333 *	0.308 *	3.909 ^NS^	3.195 **	1.988 **
H × P × T	4	0.086 ^NS^	0.063 ^NS^	2.204 ^NS^	1.091 ^NS^	2.319 *
S × P × T	6	0.074 ^NS^	0.076 ^NS^	4.375 ^NS^	0.724 ^NS^	0.403 ^NS^
H × S × P × T	12	0.039 ^NS^	0.026 ^NS^	5.063 ^NS^	0.615 ^NS^	0.474 ^NS^
Error	144	0.171	0.166	3.693	0.622	0.781

* Significant at the 0.05 level; ** Significant at the 0.01 level; NS, Not significant.

**Table 2:** Evolution of capsaicinoids and antioxidants in hot peppers under different storage conditions. Data shown are the mean values ± standard deviation for three hot pepper hybrids (Sky Red, Maha and Wonder King), and ▼ is the percent reduction from 0 to 150 days on a dry weight basis.

Parameter (mg 100 g^−1^)	Polyethylene bag				Jute bag			
	Days	20 °C	25 °C	30 °C	Days	20 °C	25 °C	30 °C
Capsaicin	0	26.9 ± 6.0			0	26.9 ± 6.0		
	50	26.0 ± 5.7	25.8 ± 6.2	25.4 ± 6.1	50	25.8 ± 5.7	25.2 ± 5.9	24.9 ± 5.9
	100	25.2 ± 6.1	24.8 ± 6.0	24.5 ± 5.5	100	25.2 ± 6.0	24.1 ± 6.1	24.1 ± 5.8
	150	24.5 ± 6.1	24.1 ± 5.1	23.4 ± 6.0	150	24.4 ± 6.1	23.6 ± 6.0	23.1 ± 6.0
	▼	8.8%	10.4%	12.9%	▼	9.2%	12.2%	14.0%
Dihydrocapsaicin	0	16.6 ± 3.6			0	16.6 ± 3.6		
	50	16.0 ± 3.8	15.5 ± 3.7	15.0 ± 3.4	50	15.8 ± 3.8	15.2 ± 3.5	15.3 ± 3.9
	100	15.4 ± 3.7	15.2 ± 3.7	14.4 ± 3.5	100	15.3 ± 3.5	14.6 ± 3.6	14.3 ± 3.6
	150	14.8 ± 3.4	14.5 ± 3.9	14.0 ± 3.6	150	14.9 ± 3.1	14.1 ± 3.7	13.9 ± 4.0
	▼	11.1%	12.8%	15.4%	▼	10.0%	14.9%	16.0%
Total carotenoids	0	95.5 ± 14.6			0	95.5 ± 14.6		
	50	91.5 ± 14.2	89.6 ± 15.0	89.4 ± 13.6	50	90.9 ± 13.8	87.9 ± 15.3	88.1 ± 12.8
	100	87.4 ± 15.6	86.3 ± 15.4	86.7 ± 12.0	100	87.3 ± 15.1	83.9 ± 14.7	83.3 ± 15.2
	150	84.6 ± 15.0	80.2 ± 16.6	76.2 ± 16.2	150	84.3 ± 15.0	78.5 ± 16.6	73.7 ± 15.5
	▼	11.5%	16.1%	20.2%	▼	11.7%	17.8%	22.9%
Ascorbic acid	0	40.3 ± 4.5			0	40.3 ± 4.5		
	50	38.2 ± 4.4	36.1 ± 3.7	35.7 ± 4.0	50	37.1 ± 4.7	35.6 ± 4.8	34.5 ± 4.1
	100	35.9 ± 4.4	34.6 ± 3.1	32.3 ± 2.8	100	35.3 ± 4.2	33.1 ± 3.9	31.4 ± 3.3
	150	35.4 ± 4.7	31.4 ± 3.0	29.2 ± 1.6	150	34.3 ± 4.1	29.7 ± 2.6	26.9 ± 3.1
	▼	12.2%	22.1%	27.6%	▼	14.7%	26.2%	33.2%
Total phenolic compounds	0	56.2 ± 13.6			0	56.2 ± 13.6		
	50	54.3 ± 13.2	52.7 ± 14.2	51.7 ± 15.2	50	53.9 ± 13.6	51.9 ± 14.5	51.2 ± 14.6
	100	51.1 ± 13.3	49.4 ± 13.5	46.9 ± 15.1	100	50.7 ± 14.4	48.1 ± 13.0	45.7 ± 14.0
	150	48.2 ± 12.8	46.7 ± 14.2	44.6 ± 14.2	150	47.6 ± 13.9	45.5 ± 15.1	43.4 ± 13.7
	▼	14.1%	16.9%	20.5%	▼	15.3%	19.0%	22.8%

### 3.2. Evolution of Total Carotenoids

**Figure 2:**
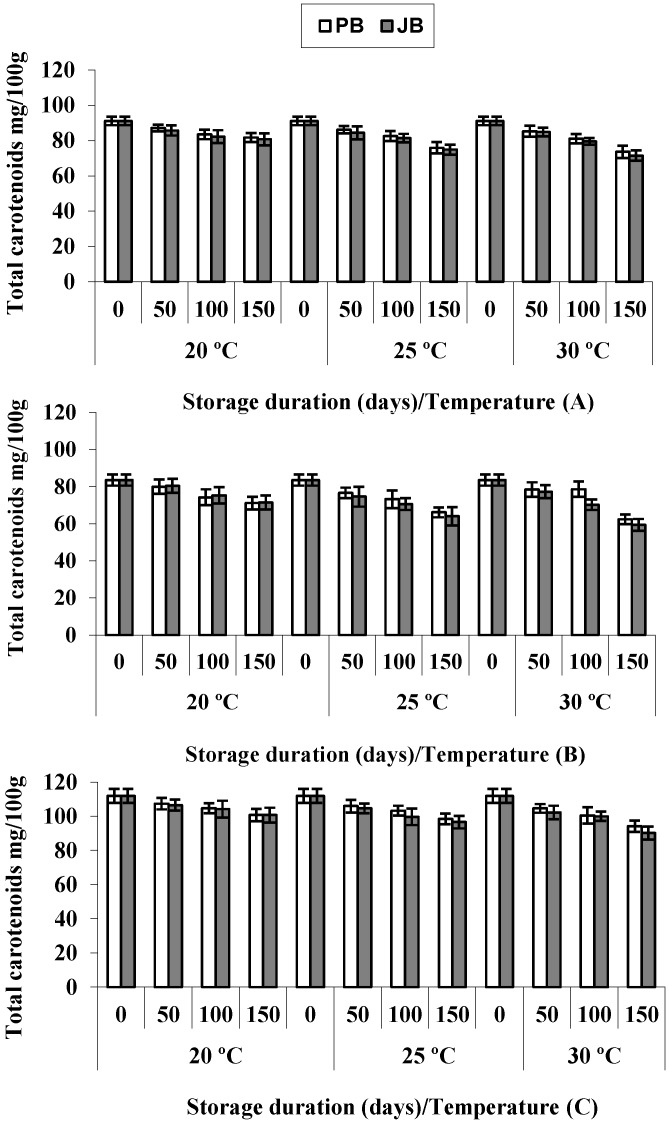
Total carotenoids in hot peppers *viz*. Sky Red (**A**), Maha (**B**) and Wonder King (**C**) at different temperatures and packaging materials during five months’ storage. Vertical bars show ± SD of *n* = 3 replicates.

Increasing interest is being paid to the red hot pepper spice, not only because of its economic importance, but also because of its diversified composition. The intense red color of the ripe peppers and their processed products is due to the presence of carotenoid pigments. When carotenoids are ingested, they show important biological actions, such as being antioxidants, as well as free radical scavengers and reducing cardiovascular diseases and the risk of several types of cancer [19,20]. Hot pepper hybrids differed significantly with respect to total carotenoid concentration (Table 1), and it was highest in Wonder King (111.8 mg/100 g), followed by Sky Red (91.2 mg/100 g), while in case of Maha, it was 83.5 mg/100 g (Figure 2). Trends observed during the storage period showed that total carotenoid concentration in hot pepper hybrids had an inverse relation with storage time (Table 2). Again, carotenoids were significantly reduced during storage for 150 days (*p <* 0.05), and the rate of disappearance increased with storage temperature and tended to be slightly higher in peppers packed in jute bags than in polyethylene. The gradual fading out of total carotenoid concentrations that occurred during storage amounted to 11.5%–20.2% in samples stored in polyethylene bags and from 11.7% to 22.9% in those packed in jute bags. The decreasing rate of total carotenoids was higher between 100 to 150 days at 30 °C, especially in the Maha hybrid (Figure 2). However, the percent reductions of carotenoids during three months’ storage were far from what could be considered as the shelf life of the product, established herein as 50% of loss. The highest reductions of carotenoids were observed in the Maha hybrid, followed by Sky Red and Wonder King, which is another example of the influence of genotype on the content of bioactive compounds in red peppers [21].

As carotenoids are thermally sensitive in nature, their disappearance rates were higher at 30 °C than at 20 °C or 25 °C. Likewise, Lee *et al*. [22] showed that carotenoid destruction in red peppers was greatly affected by water activity and storage temperature and reported that carotenoid stability in red peppers was improved by lowering the storage temperature. Carotenoids are fairly stable in their natural environment, but become sensitive to light and temperature and are readily decomposed during some food processing operations [13]. Our results are in line with the findings of Schweiggert *et al*. [23] that total carotenoid concentration during four months’ storage at ambient temperature dropped by 9.6% and 16.7% in chili and 38.8% and 39.7% in paprika powders with and without illumination, respectively. Similarly variation or loss of carotenoids during foods processing, e.g., peeling, as well as storage, occurs through geometric isomerization and enzymatic or non-enzymatic oxidation [24]. It has been reported that the loss of the carotenoid fraction during storage correlates with the development of prooxidative processes in paprika [13].

### 3.3. Evolution of Ascorbic Acid 

**Figure 3:**
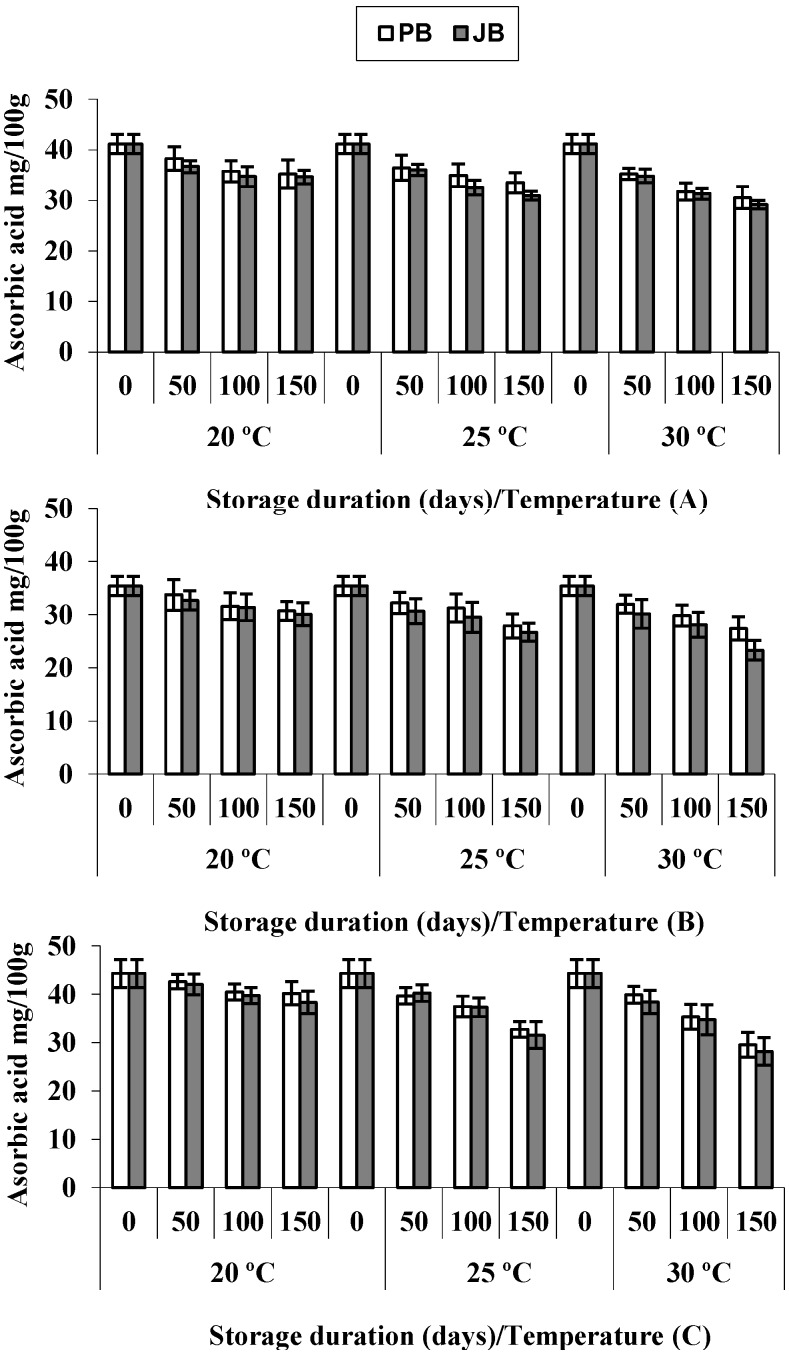
Ascorbic acid in hot peppers *viz*. Sky Red (**A**), Maha (**B**) and Wonder King (**C**) at different temperatures and packaging materials during five months’ storage. Vertical bars show ± SD of *n* = 3 replicates.

Ascorbic acid is not only well known as being an antioxidant and biologically-active compound, but also as an important nutritional and functional constituent of hot pepper fruit. Changes in the pattern of ascorbic acid concentration during five months’ storage showed that as storage time prolonged, the ascorbic acid concentration in hot peppers decreased significantly (*p* ≤ 0.05) (Table 1). The general tendency for the amount of ascorbic acid that was lost was similar for all samples and showed a total retention of 87.8%, 77.9% and 72.4% (20 °C, 25 °C and 30 °C, respectively) in polyethylene bags and 85.3%, 73.8% and 66.8% (20 °C, 25 °C and 30 °C, respectively) in jute bags, after 150 days of storage (Table 2). Then, the ascorbic acid concentration gradually decreased in hot pepper hybrids during storage, and the disappearance rate increased with storage temperature and tended to be higher in jute bags, reaching a maximum of 33.2%. A steep decrease in ascorbic acid concentrations was observed until Day 100, especially in the Wonder King hybrid, but further reduction still occurred between 100 and 150 days of storage (Figure 3).

These results revealed that ascorbic acid is sensitive to high temperatures during storage, and it gradually decreased in all hot pepper hybrids. Similarly, Kalt [12] indicated that ascorbic acid concentration decreased in many major fruits and vegetables during storage. Daood *et al*. [11] reported that ascorbic acid concentration in ground paprika decreased by 10% after 30 days followed by 20% and 35% after 60 and 120 days of storage. It has been reported that modified atmosphere packaging (MAP) can retard the decline of ascorbic acid in intact peppers, as Ornelas-Paz *et al*. [25] observed that its contents remained unchanged in peppers stored under MAP during four weeks.

### 3.4. Evolutions of Total Phenolic Compounds 

**Figure 4:**
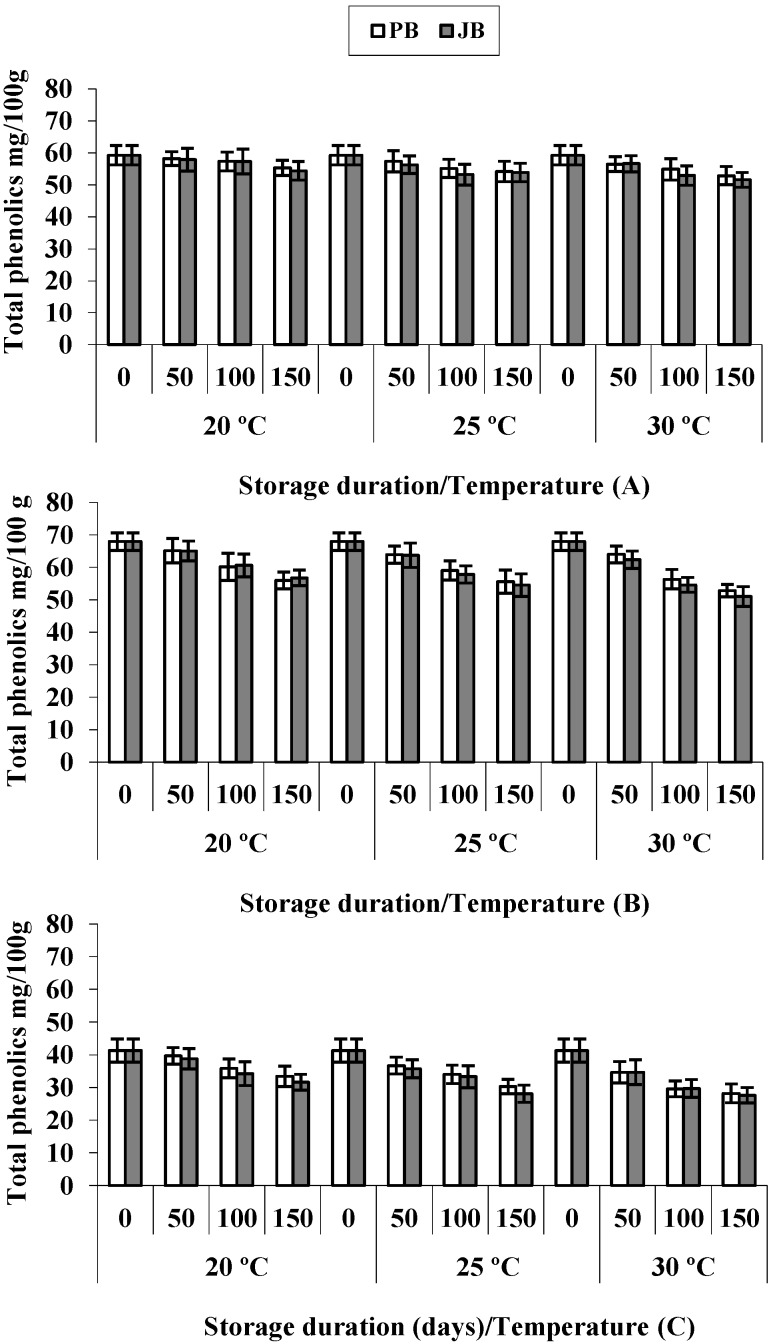
Total phenolic contents in hot peppers *viz*. Sky Red (**A**), Maha (**B**) and Wonder King (**C**) at different temperatures and packaging materials during five months’ storage. Vertical bars show + SD of *n* = 3 replicates.

Besides the antioxidant activity of phenolic compounds that can protect the human body from free radicals, these compounds also possess antimicrobial properties [26], and they are natural inhibitors of the growth of toxigenic *Aspergillus* and aflatoxin B_1_ production [27], which is a significant risk in hot peppers [28]. Table 1 shows that hot pepper hybrids differed significantly (*p* ≤ 0.05) with respect to their total phenolic contents, with Maha standing first (67.9 mg/100 g), followed by Sky Red (59.3 mg/100 g) and Wonder King (41.2 mg/100 g) (Figure 4). Total phenolic concentrations decreased continuously in polyethylene-packed hot peppers during 150-day storage by 14.1% at 20 °C, 16.9% at 25 °C and 20.5% at 30 °C, while decreasing amounts in samples packed in jute bags were 15.3%, 19.0% and 22.8%, respectively (Table 2). Then, total phenolic compounds declined significantly during storage for 150 days (*p <* 0.05), while the disappearance rate increased with storage temperature. The trends of phenolics evolution in the three hot pepper hybrids is illustrated in Figure 4. Regarding the stability in the different packing materials, the total phenolic contents followed the same trend in polyethylene and jute-packed peppers, indicating that both packaging materials preserved these compounds to a similar extent. 

The present studies are in line with the findings of Talcott *et al.* [8] that the fading out of phenolic acids was accentuated by storage at high temperatures and affected the antioxidant activity. Similarly, Mustapha and Selselet-Attou [9] reported that phenolic compounds in dates decreased gradually during storage for five months, decreasing by 24% at ambient temperature and by 14% at 10 °C.

It has been reported that MAP can retard the ripening process in peppers. Ornelas-Paz *et al*. [25] observed that phenolic contents were not reduced in peppers stored under MAP during four weeks.

## 4. Conclusions

In light of the present results, it can be concluded that with the increase in storage temperature and time, a significant, but gradual decrease in capsaicinoids and antioxidant levels occurred in dry whole peppers. However, despite the sensitivity of capsaicinoids and antioxidants to oxygen, light and moisture, the packaging in natural jute bags or synthetic LDPE plastic bags, as well as the storage at ambient temperature preserved between 77.4% and 87.3% of the initial amounts of these health and nutrition-promoting compounds during five months’ storage. Therefore, the traditional sun-drying of pepper fruits and the application of commercially-controlled conditions of storage is able to maintain the overall quality of peppers in terms of pungency, color and antioxidants.
